# 融合MassWorks质谱解析技术与气相色谱-低分辨质谱法的本科教学创新实践

**DOI:** 10.3724/SP.J.1123.2025.11005

**Published:** 2026-07-08

**Authors:** Zhenxing LI, Yu’an SUN, Bin LI, Wenhao YU, Weimin OUYANG, Yachang XU, Kuan TIAN, Jianbo ZHAO

**Affiliations:** 1. 郑州轻工业大学，河南 郑州 450001; 1. Zhengzhou University of Light Industry，Zhengzhou 450001，China; 2. 郑州轻大产业技术研究院有限公司，河南 郑州 450001; 2. Zhengzhou Qingda Industrial Technology Research Institute Co. ，Ltd. ，Zhengzhou 450001，China; 3. 北京绿绵科技有限公司，北京 100080; 3. Beijing Green Cotton Technology Co. ，Ltd. ，Beijing 100080，China

**Keywords:** MassWorks, 气相色谱-低分辨质谱法, 教学改革, 分子式识别, 同位素谱图准确度, MassWorks, gas chromatography-low resolution mass spectrometry, teaching reform, molecular formula identification, accuracy of isotopic spectrum

## Abstract

针对质谱应用人才短缺的行业现状与本科教学中仪器资源有限、学生定性能力薄弱、原理理解困难等瓶颈，开展了一项基于常规气相色谱-质谱法与MassWorks专业质谱解析软件相结合的教学改革实践。以14种香料混合标准品为分析对象，实验设计覆盖分离、检测、精确质量测定、同位素分布匹配、分子式识别、结构解析全流程，借助软件的精确质量校准与同位素峰形校准双校正技术，显著提升了低分辨质谱数据的质量精度与定性可靠性。实践结果显示，该方法使质谱数据质量精度提升100倍，质量偏差控制在|Δ*m|*≤10 mDa，同位素谱图准确度达98%以上，定性结果准确可靠，教学成效显著。通过理论解析、软件应用、实践验证的闭环训练，有效培养了学生的科研思维、数据分析能力及解决复杂问题的创新能力，为在有限资源条件下提升仪器分析教学质量提供了可复制、可推广的有效范式。

## Abstract (en)

Mass spectrometry professionals are in short supply industry-wide. Undergraduate teaching has bottlenecks： limited instrumentation， weak qualitative skills and abstract theoretical concepts. We report an innovative pedagogy coupling conventional gas chromatography-mass spectrometry （GC-MS） with the MassWorks^TM^ software suite. A 14-component fragrance standard mixture was employed to design an experiment spanning the entire workflow separation， detection， exact-mass measurement， isotopic-pattern matching， molecular-formula assignment， structural elucidation. By exploiting the software’s dual-calibration algorithms for exact-mass and isotopic-profile refinement， the mass accuracy and qualitative reliability of GC-low resolution MS data were dramatically enhanced. The practical results show that this method improves the quality accuracy of MS data by 100 times， controls the mass deviation within |Δ*m*|≤10 mDa， achieves an accuracy of over 98% in isotope spectra， and provides accurate and reliable qualitative results. The teaching effect is significant. Through the closed-loop training of theoretical analysis， software application， and practical verification， students’ scientific research thinking， data analysis ability， and innovative ability to solve complex problems have been effectively improved. This provides a replicable and scalable effective paradigm for improving the quality of instrument analysis teaching under limited resource conditions.

质谱分析技术作为揭示物质组成的关键工具，被誉为现代科学研究的“眼睛”，在环境科学、生命科学及药物研发等领域具有不可或缺的作用^［1，2］^。据2020-2023年中国质谱仪器市场调研报告显示，质谱类设备年采购量约10 000台，年销售额突破百亿元^［3］^。2025年质谱人才发展论坛相关研讨数据显示，随着质谱仪器市场保有量的快速攀升，兼具仪器原理认知、行业应用经验与专业数据分析能力的复合型人才供给缺口持续扩大。当前行业尚未形成标准化、系统化的人才培养体系，这种人才储备的短板，正逐步成为限制质谱领域规模化、高质量发展的核心因素。

面对挑战，高校教育在人才培养方面的作用愈发凸显，培养具备多学科交叉与创新能力的专业人才，既契合学生成长的内在需求，也顺应社会发展对高素质人才的迫切需要^［4，5］^。近年来，质谱教学在理工科教育中地位不断提升，诸多学者也开展了相关教学实践探索^［6-8］^。本科阶段开设未知物结构鉴定的实验课程，有助于培养学生综合运用理论知识解决实际问题的能力^［9］^，但高分辨质谱仪因购置与维护成本高且操作复杂，在本科实验教学中普及度极低^［10，11］^，大多数高校主要采用气相色谱-低分辨质谱仪（GC-LRMS）开展仪器分析实验教学。

GC-LRMS虽然能够提供化合物的保留时间与质谱碎片信息，但其质量精度通常为±0.5 Da，难以实现精确质量测定与分子式识别等核心定性环节，导致教学过程高度依赖商业谱库检索。该现象致使学生对质谱定性的理解停留在保留时间比对和谱图匹配等浅层操作层面，无法建立从原始数据出发，通过精确质量分析、同位素分布评估到结构推导的完整逻辑链条，限制了其科研创新能力的系统培养。

近些年，质谱数据处理技术的发展为突破上述教学瓶颈提供了新的可能。Cerno Bioscience公司开发的MassWorks质谱解析软件，基于全氟三丁胺（PFTBA）等标准物质，采用精确质量校准与同位素峰形校准双重校正策略^［12-14］^，显著提升低分辨质谱数据的质量精度（可达±0.005 Da），并利用软件把实验测得的同位素峰簇整体形状（包括同位素峰的相对强度、峰形宽度和峰位偏移）与理论计算得到的最有可能分子式的同位素分布进行最小二乘法拟合，给出0~100%的量化指标同位素谱图准确度辅助分子式识别，有效增强低分辨数据的定性可靠性^［15-18］^。因此，该研究将MassWorks软件与高校广泛配置的GC-LRMS相结合，设计并实施面向本科生的综合创新实验。通过引导学生完成从样品分析、数据校正到结构确证的全流程训练，探索在有限硬件资源条件下深化质谱教学内容、强化学生科研思维与创新能力的有效途径，旨在为仪器分析及相关专业的实验教学改革提供可借鉴的实践范例。

## 1 教学改革设计思路

针对教学过程中学生对精确质量、同位素分布等抽象概念认知脱节，解决实际问题能力断层及高分辨仪器匮乏，低分辨仪器功能未被充分发掘的教学痛点，利用MassWorks质谱解析软件作为数据解析增强器和思维引导工具，本研究构建了“低分辨仪器+高级软件+综合实验设计”的三维教学模式，将单一谱图检索实验升级为完整的定性分析全流程，贯彻教育部提出的“以学生为中心、产出为导向、持续改进为目标”的教学理念，推动高等教育高质量发展。

## 2 实验部分

### 2.1 仪器与试剂

Agilent 7890A-5975C气相色谱-质谱联用仪（美国Agilent公司）；MassWorks 7.0质谱解析软件（美国Cerno Bioscience 公司）；14种混合香料标样（含香茅醇、香叶醇、芳樟醇、柠檬烯、蒎烯等，天津阿尔塔科技有限公司）；甲醇（色谱纯，德国默克公司）。

### 2.2 仪器参数

色谱条件 HP-5 MS色谱柱（30 m×0.25 mm×0.25 μm）；程序升温：初始温度50 ℃，保持4 min，以8 ℃/min升至160 ℃，保持5 min；恒定流量模式，载气为高纯氦气（纯度≥99.999%），流速为1.2 mL/min；进样口温度260 ℃；分流比50∶1。

质谱条件 电子轰击（EI）离子源，电子能量70 eV；离子传输线温度250 ℃；离子源温度230 ℃；四极杆温度150 ℃；溶剂延迟3 min；全扫描profile采样模式，质量扫描范围*m/z* 40~450；PFTBA标准品为质谱外标校正液。

### 2.3 实验流程及安排

本课程授课人数30人，采取理论与实验相结合的教学方式，理论课程采取集中讲解，实验部分实行分组轮转，每组10人，以增加学生实操能力，课程安排共12学时。理论课程部分包括气相色谱-质谱联用仪的发展史、仪器构造和工作原理、MassWorks软件操作规程等（2学时）和精确质量数、分辨率、同位素丰度、分子式识别、谱图准确度的定义和应用讲解（1学时）。实验部分包括学生独立操作GC-MS，获取混合样品的总离子流图（TIC）和各组分的质谱图（2学时）；在相同测试条件下采集PFTBA的质谱图，导入MassWorks软件，选择特征离子（*m/z* 69、*m/z* 131、*m/z* 219等）建立质量和峰形校正文件（1学时）；将校正文件应用于混合样品数据处理，获取校正后的精确质量数，利用软件的分子式查找功能，生成候选分子式列表，通过比较理论同位素分布与校正后实测同位素谱图准确度，筛选出最可能的分子式（2学时）；对分子离子和主要碎片离子进行分子式归属，结合碎片规律和不饱和度，讨论化合物的可能结构，开展课堂提问、同伴互评、进行实验改进和提升趣味性讨论等环节；最后，撰写实验报告，重点阐述定性分析的逻辑过程和数据依据（4学时）。

## 3 结果与讨论

### 3.1 精确质量和同位素峰形校正

采用PFTBA特征离子为校准对象，通过双维度校正策略完成质谱系统校准。选取具备差异化质量分布特征的离子原始数据，导入MassWorks数据分析软件开展校正操作。首先，以已知元素组成的碎片离子为校准基准，将其实测质荷比与理论精确质量进行拟合，完成特定质量区间内的质量轴精准校准；同时，以目标碎片离子对应理论分子式的同位素峰形与丰度分布为参照标准，对实测碎片离子的峰形参数进行拟合优化。最终整合质量轴校准与峰形参数拟合得到的最优参数，构建得到适用于该检测体系的质量峰形校正函数文件，为后续质谱数据的精准定性与定量分析奠定基础。参与校正的碎片离子信息如表1所示。

**表 1:** 全氟三丁胺的特征碎片离子校正信息

No.	Net formula	Mono isotopic mass	Closest centroid	Δ*m/*mDa	Δ*m*/*M （*10^-6^）	Spectral accuracy/%
1	CF_3_^+^	68.9947	68.9947	0.0057	0.1	99.8
2	C_2_F_5_^+^	118.9915	118.9915	0.0137	0.1	99.9
3	C_3_F_5_^+^	130.9915	130.9921	0.6733	5.1	99.9
4	C_3_F_7_^+^	168.9883	168.9890	0.7392	4.4	99.8
5	C_4_F_9_^+^	218.9851	218.9869	1.7779	8.1	99.9
6	C_5_F_10_N^+^	263.9866	263.9883	1.7355	6.6	99.9
7	C_8_F_16_N^+^	413.9770	413.9761	-0.8562	-2.1	99.8

Δ*m*： the absolute value of the difference between the theoretical *m*/*z* of target ion and the test *m*/*z*； *M*： theoretical *m*/*z* of target ions.

图1为全氟三丁胺特征碎片离子校正前后谱图优化对比图，结合表1校正结果信息综合分析，PFTBA参与校正的7个特征碎片离子校正后质量与理论质量偏差均小于1.8 mDa，同位素谱图准确度均在99%以上，满足MassWorks软件对质谱校正函数绝对质量偏差小于5 mDa，校正后同位素谱图准确度大于98%的要求，为低分辨质谱获取高质量精度数据和多维定性分析奠定基础。

**图 1:**
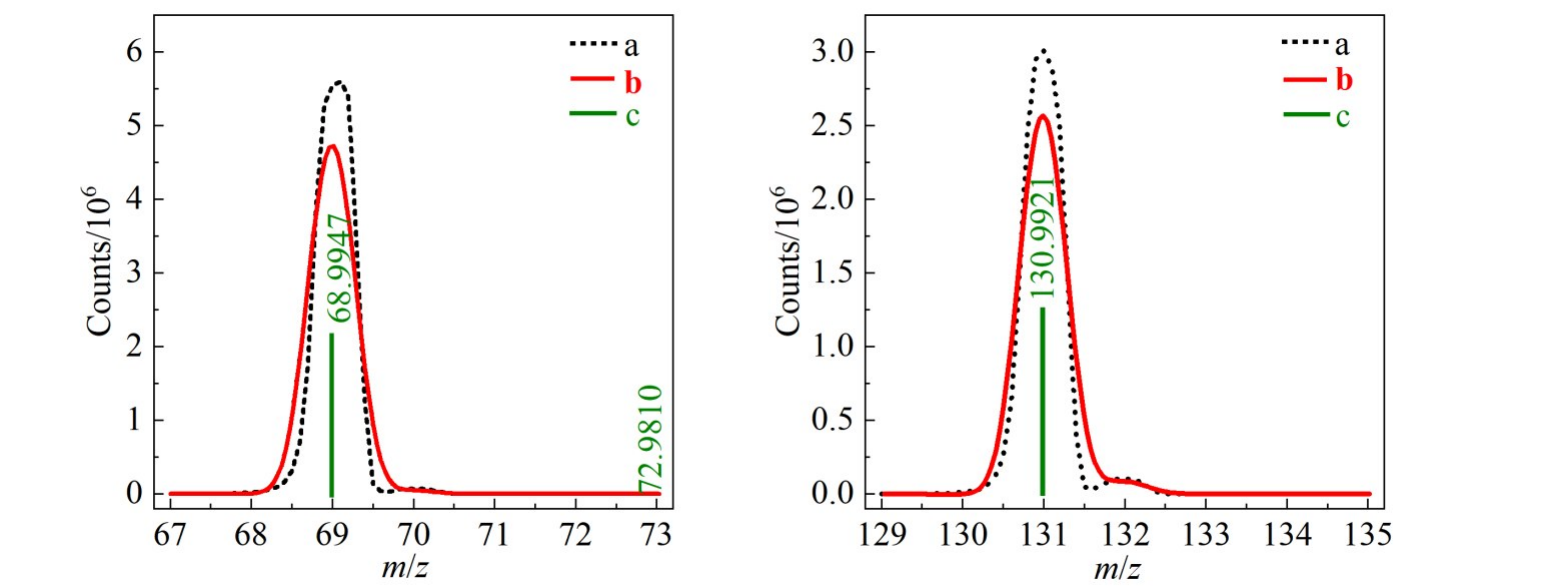
全氟三丁胺部分特征碎片离子校正结果

### 3.2 香料样品的分子式预测与识别

学生借助气相色谱-质谱联用仪开展实操训练，通过优化色谱柱类型、柱温程序、载气流速等核心分离条件，完成对14种香料混合物分离，并获得其总离子流图（见图2），谱图中各组分实现基线分离，满足实验教学测试需求。利用MassWorks软件，导入质谱校正文件，对谱图中选定化合物峰进行校正操作，即可得到该物质所有离子的精确质量数信息。

**图2:**
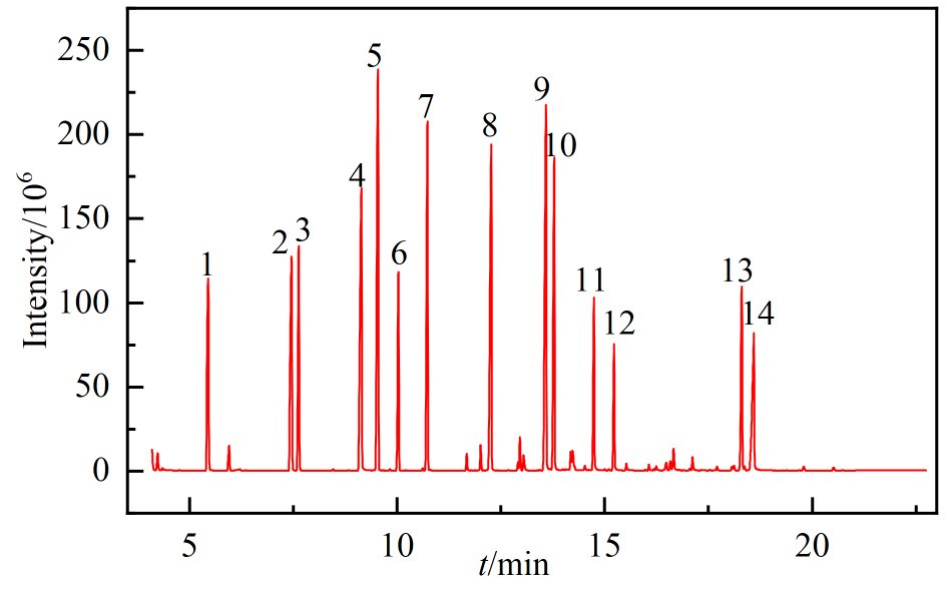
香料混合物的总离子流图

#### 3.2.1 分子式预测与同位素匹配

目前高分辨质谱软件中，分子式预测主要依赖精确相对分子质量测定，其原理类似解多元一次方程。假设实测分子质量为*M*，质量偏差范围为 ∆m ，若化合物含C、H、O这3种元素，原子个数分别为*X*、*Y*、*Z*，则预测公式见式（1）：


M±∆m=X×12+Y×1.0078+Z×15.9949 (1)

经过公式计算，结果存在多个候选分子式。如何在候选分子中快速锁定正确结构，成为影响科研进展的重要因素。MassWorks软件可模拟分子式的理论质谱同位素分布，通过对比测试样品分子式与预测分子式同位素分布拟合程度，提升预测结果的准确性。同位素分布拟合谱图准确度越高，分子式的合理性越强。

实验以18.3 min对应的α-紫罗酮分子识别为例，演示MassWorks软件对化合物分子识别功能的应用。谱图测试显示化合物精确质量为192.152 2 Da，在Δ*m*为20 mDa范围内，通过计算得到3个候选分子式C_13_H_20_O、C_10_H_24_O_3_、C_9_H_20_O_4_，并对3个分子式理论质谱图和测试质谱图进行比较，如图3所示。由于3个分子理论精确分子质量（取小数点后4位）均为192.152 2 Da，很难对预测分子式进行确认，只能选择标准品或其他检验进行验证；但是利用 MassWorks软件引入谱图准确度进行判断，显示α-紫罗酮的正确分子式C_13_H_20_O的谱图与测试质谱图准确度达99.61%，理论分子式同位素谱图与校正后样品谱图完美重合，其余两个分子式的谱图准确度分别为97.35%和96.44%，理论同位素分布与样品谱图差异显著，可快速有效排除干扰。

**图3:**
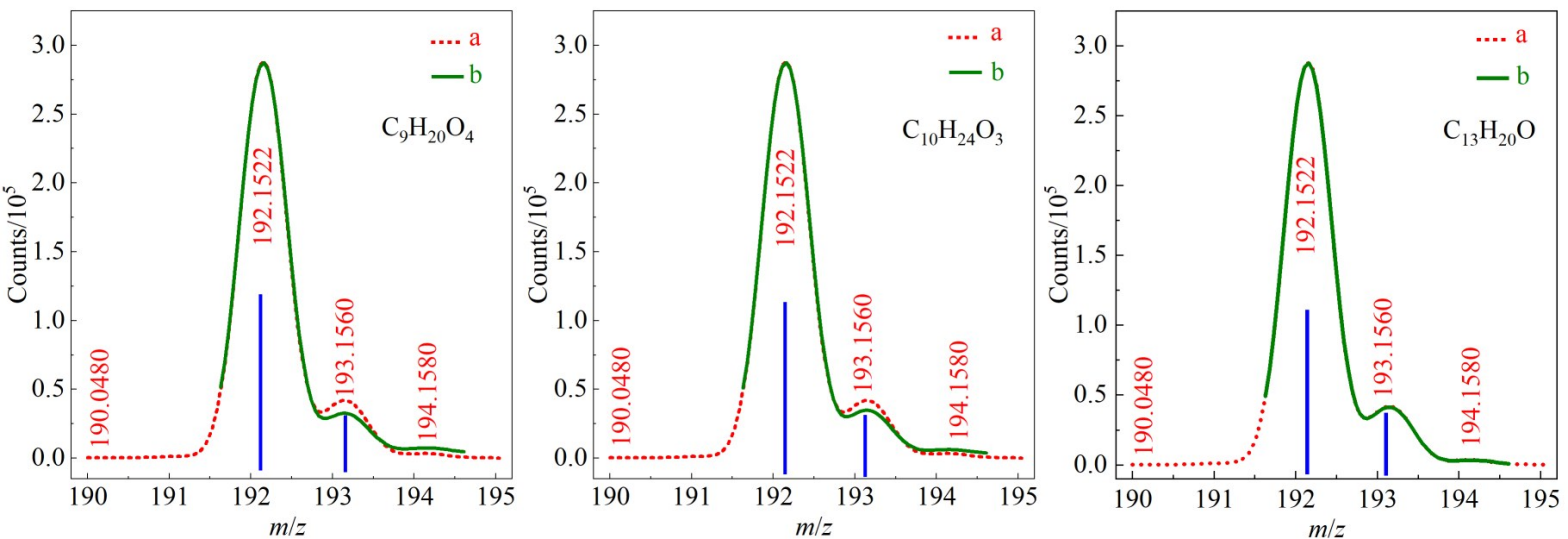
**α**-紫罗酮对应预测分子式同位素谱图准确度

#### 3.2.2 香料组分的分子式识别

按照精确质量数和谱图准确度校正操作，对谱图中14种化合物分子式进行确认，结果及相关信息如表2所示。数据表明，在14种香料化合物定性分析过程中，其中13种化合物经软件质量精度校正后，与理论质量偏差均在|Δ*m*|≤10 mDa范围内，说明软件对低分辨质谱质量精度提升效果明显；只有薄荷醇一种化合物质量偏差为22.67 mDa，经过对比分析研判，发现该物质的分子离子峰响应微弱，信号过低，导致校正偏差增大，主要由化合物本身结构性质决定，可适当提升化合物浓度，提升信号响应，降低校正误差来改善质量精度。

**表 2:** 香料样品经MassWorks软件校正后的分子式识别结果

No.	*t*/min	Compound	Molecular formula	Theoretical quality	Calibration quality	Δ*m/*mDa	Spectral accuracy/%
1	5.95	isovaleraldehyde	C₅H₁₀O	86.0726	86.0639	-8.72	98.48
2	7.45	cyclohexanone	C₆H₁₀O	98.0726	98.0793	6.68	99.43
3	7.62	ethyl valerate	C₇H₁₄O₂	130.0998	130.0898	-9.03	98.97
4	9.13	benzaldehyde	C₇H₆O	106.0413	106.0357	-5.62	98.87
5	9.53	*β*-pinene	C₁₀H₁₆	136.1247	136.1239	-0.75	99.81
6	10.03	*n*-decane	C₁₀H₂₂	142.1716	142.1698	-1.8	99.73
7	10.72	D-limonene	C₁₀H₁₆	136.1247	136.1222	-2.45	99.79
8	12.26	linalool	C₁₀H₁₈O	154.1352	154.1299	-5.32	99.33
9	13.58	benzyl acetate	C₉H₁₀O₂	150.0675	150.0758	8.27	99.66
10	13.78	menthol	C₁₀H₂₀O	156.1509	156.1282	-22.67	98.43
11	14.74	citronellol	C₁₀H₂₀O	156.1509	156.1414	-9.64	99.88
12	15.22	geraniol	C₁₀H₁₈O	154.1352	154.1274	-7.82	99.74
13	18.3	α-ionone	C₁₃H₂₀O	192.1509	192.1522	1.33	99.61
14	18.59	coumarin	C₉H₆O₂	146.0362	146.0322	-4.03	99.26

#### 3.2.3 分子式结构推断

借助MassWorks软件精确质量数和同位素谱图准确度双校正技术，可以高效识别化合物分子式，由于元素组成相同而结构不同的同分异构体众多，同样无法获取化合物准确结构。为了解决上述问题，利用MassWorks软件质量校正功能，根据化合物碎片精确质量识别对应元素组成，结合元素组成的不饱和度及分子式断裂特征丢失规律，按图索骥，推断出分子离子峰和特征碎片离子结构，同时也可通过碎片离子验证疑似结构的合理性。

通常在有机化合物结构推断过程中，不饱和度（*Ω*）是重要的参考指标。假设待分析化合物组成为C *_x_* H *_y_* O *_z_* ，分子式不饱和度计算公式为*Ω=x+*1-*y/*2*。*

当化合物中含有卤素原子（-X）、硝基（-NO_2_）、氨基（-NH_2_）等，可采用等效替换原则，将此类官能团仅作氢原子等效计算。不饱和度为1时，表明化合物含有1个双键或1个环；不饱和度为2时，化合物可能含有1个三键或2个双键，或一个双键和一个环，以此类推，能够验证化合物结构信息。

以芳樟醇质谱碎片为例向学生进行分子式结构推断过程讲解。根据分子离子峰信息确认分子式为C_10_H_18_O，经计算化合物不饱和度为2，显示分子中含有两个不饱和键；碎片离子*m/z* 139.109 1与分子离子*m/z* 154.127 4相差15.018 3 Da，通过元素匹配两者相差元素组成为CH_3_，质谱峰对应［M-CH_3_］^+^，同理碎片离子*m/z* 136.122 0、*m/z* 121.102 7、*m/z* 93.082 8对应分别为［M-H_2_O］^+^、［M-CH_3_-H_2_O］^+^、［M-C_3_H_7_-H_2_O］^+^，同时所对应元素组成不饱和度均大于2，说明脱落碎片均为饱和键，与精确质量数推断结果一致。而碎片离子中质谱峰*m/z* 83.008 1和*m/z* 71.070 3，经过精确质量数预测匹配，对应元素组成分别为C_6_H_11_和C_4_H_7_O，两者不饱和度均为1.5，说明两个基团中分别含有一个不饱和键。结合美国国家标准与技术研究院质谱中心（NIST）谱库检索信息，最终确认C_6_H_11_（*m/z* 83）化合物结构为（CH_3_）_2_C=CH-CH_2_-CH_2_-；由于C_10_H_18_O既能产生脱甲基和脱水碎片，推理可知C_4_H_7_O（*m/z* 71）结构为HC=CH-（CH_3_）CH（OH）-，详细信息见表3。

**表 3:** 基于芳樟醇碎片离子精确质量识别分子式结果

No.	Molecular formula	Precision accuracy	Δ*m/*mDa	Spectral accuracy	Degree of unsaturation	Fragmented relationship
1	C₁₀H₁₈O	154.1274	-7.82	99.74	2	M *^+^*
2	C₉H₁₅O	139.1091	-2.64	99.61	2.5	［M-CH₃］⁺
3	C₁₀H₁₆	136.1220	-2.65	99.54	3.0	［M-H₂O］⁺
4	C₉H₁₃	121.1027	1.52	99.83	3.5	［M-CH₃-H₂O］⁺
5	C₇H₉	93.0828	12.92	99.17	3.5	［M-C₃H₇-H₂O］⁺
6	C₆H₁₁	83.0881	2.57	99.36	1.5	［M-C₄H₇O］⁺
7	C₄H₇O	71.0703	21.16	99.23	1.5	［M-C₆H₁₁］⁺

## 4 教学成效分析

### 4.1 数据质量显著提升

采用MassWorks校正技术，GC-LRMS的数据质量实现了质的飞跃。混合样品中14种化合物的分析结果显示，绝大多数化合物的质量偏差被控制在|Δ*m*|≤10 mDa范围内，同位素谱图准确度均高于98%。这使得利用低分辨的仪器获得接近高分辨数据的定性能力成为可能，有效解决了教学中的技术瓶颈。

### 4.2 学生能力全面提升

软件的可视化界面使学生能够直观地观察质谱图在校准前后的变化，将抽象的精确质量与同位素丰度等概念具象化，从而加深学生对理论知识的理解。在此过程中，学生学习并掌握了科研中广泛使用的专业质谱解析工具，有效提升了实际操作能力与就业竞争力。同时，整个实验设计突破了传统“照方抓药”的模式，引导学生以科研人员的思维方式展开探索，包括如何评估数据质量、如何从多个候选方案中确定最优解，以及如何利用碎片信息验证假设。这一过程完整模拟了提出问题、分析问题、解决问题的科学研究逻辑，系统培养了学生的科研思维与综合问题解决能力。

### 4.3 教学反馈积极

通过课程问卷和访谈发现，学生普遍对该实验项目评价较高。他们认为：“实验方式新颖，参与感强且具有挑战性，需通过亲身体验获得实验结果，成就感满满”“第一次感觉自己真正在解析质谱图”，而不是单纯地“看结果”。专业指导教师也反馈，学生的参与热情和报告质量较传统实验有明显提升。

## 5 创新点与推广价值

### 5.1 主要创新点

本教学实践构建了低成本硬件借助高端软件与综合设计实验相结合的新模式，实现了教学资源的高效整合与最优配置。实验内容上，将质谱数据处理中的前沿技术——谱图准确度评估引入本科实验教学，拓展了教学内容的深度与前沿性。在方法上，通过将PBL（问题驱动学习）教学法融入仪器分析实验，推动教学重心由传统技能训练向综合能力培养的实质性转变，有效提升了学生的科学思维与创新素养。

### 5.2 推广价值

该教学方案所需的GC-MS是高校化学类实验室的常规配置，MassWorks软件作为商业软件，引入门槛远远低于购置高分辨仪器。因此，该模式具有极强的可复制性和推广性，易于在其他高校的分析化学、药物分析、环境监测等相关课程的实验教学中推广应用，对于提升全国范围内相关专业的人才培养质量具有积极意义。后续实验过程中还可将含有不同组分的香料样品进行编号，学生通过随机抽签进行盲样定性定量分析，进一步增加实验的复杂性和趣味性。

## 6 结语与展望

MassWorks质谱解析软件与低分辨率GC-MS相结合，成功地将一个常规的验证性实验改造为一个富有挑战性和探索性的综合创新实验。这一教学改革实践表明，通过引入先进的数据处理方法和优化实验教学设计，在不依赖昂贵硬件更新的前提下，显著提升仪器分析实验教学的质量和水平。它不仅使学生掌握了实用的分析技能，更重要的是培养了其批判性思维和解决复杂问题的创新能力，契合新时代对高素质复合型人才培养的要求。未来，可考虑将该模式拓展至LC-MS数据的处理，并尝试应用于毕业设计或科研训练中更复杂的实际样品分析，进一步深化教学效果。

## References

[1] Gu C S， Liu Z Z， Jin H L， et al . Chinese Journal of Chromatography， 2025， 43（6）： 650 40394744 10.3724/SP.J.1123.2024.08015 PMC12093205

[2] Li B， Chen S J， Chang Y Y， et al . Journal of Analytical Testing， 2025， 44（9）： 1822

[3] Analytical Instrumentation Branch of the China Instrumentation and Control Society . 2020-2023 China Mass Spectrometry Instrument Market Research Brief. （2024-01-31）［2025-09-10］. https：//fxxh.cis.org.cn/news/2498.html

[4] Zhou L， Li M， Huang Z Q， et al . Laboratory Research and Exploration， 2023， 42（1）： 235

[5] Wei Q， Zhao L， Liu S R， et al . Advances in Education， 2025， 15（1）： 1332

[6] Xu Y， Jiang L Y， Yang S S . Chinese Journal of Chromatography， 2025， 43（11）： 1268 41200975 10.3724/SP.J.1123.2025.03003 PMC12598550

[7] Lin Z A， Jin Y S . University Chemistry， 2024， 39（11）： 327

[8] Shao W， Zhang W Q， Hu W Q， et al . University Chemistry， 2025， 40（2）： 97

[9] Shen Y Q， Sun S X， Chi Q， et al . Modernization of Education， 2019， 6（53）： 62

[10] Zhu Y Y， Feng L . Laboratory Research and Exploration， 2025， 44（7）： 177

[11] Li W， Wang L， Li H Y， et al . Guangdong Chemical Industry， 2024， 51（18）： 195

[12] Adam G， Wang Y D . Proceedings of the 73rd Annual Conference on Mass Spectrometry in the United States. Baltimore： American Chemical Society， 2025： 474

[13] Emmanuel E， Killian B . Anal Chem， 2017， 89（18）： 9805 28768103

[14] Wang Y D， Gu M . Anal Chem， 2010， 82： 7055 20684651 10.1021/ac100888b

[15] Qi A A， Ma L F， Lei C N， et al . Journal of Mass Spectrometry， 2019， 40（2）： 167

[16] Hu G J， Zhou W， Zhang Y H， et al . Food Industry Technology， 2015， 36（18）： 70

[17] Liu Z F， Li B， Xu J Z， et al . Journal of Mass Spectrometry， 2014， 35（6）： 54

[18] Li W J， Yuan Y R， Ouyang W M， et al . Modern Instruments， 2010， 16（5）： 11

